# Chromosome-level Genome Assembly and Annotation of the Arctic Moss *Ptychostomum knowltonii*

**DOI:** 10.1093/gbe/evae268

**Published:** 2024-12-10

**Authors:** Changling Ma, Xuping Zhou, Dan Huang, Li Zhang, Yifeng Yao, Yang Liu, Shanshan Dong, Tao Peng

**Affiliations:** School of Life Sciences, Guizhou Normal University, Guiyang 550025, China; Key Laboratory of Southern Subtropical Plant Diversity, Fairy Lake Botanical Garden, Shenzhen & Chinese Academy of Sciences, Shenzhen 518004, China; Key Laboratory of Southern Subtropical Plant Diversity, Fairy Lake Botanical Garden, Shenzhen & Chinese Academy of Sciences, Shenzhen 518004, China; School of Life Sciences, Guizhou Normal University, Guiyang 550025, China; Key Laboratory of Southern Subtropical Plant Diversity, Fairy Lake Botanical Garden, Shenzhen & Chinese Academy of Sciences, Shenzhen 518004, China; Key Laboratory of Southern Subtropical Plant Diversity, Fairy Lake Botanical Garden, Shenzhen & Chinese Academy of Sciences, Shenzhen 518004, China; State Key Laboratory of Plant Diversity and Specialty Crops, Institute of Botany, Chinese Academy of Sciences, Beijing 100093, China; China National Botanical Garden, Beijing 100093, China; Key Laboratory of Southern Subtropical Plant Diversity, Fairy Lake Botanical Garden, Shenzhen & Chinese Academy of Sciences, Shenzhen 518004, China; Key Laboratory of Southern Subtropical Plant Diversity, Fairy Lake Botanical Garden, Shenzhen & Chinese Academy of Sciences, Shenzhen 518004, China; School of Life Sciences, Guizhou Normal University, Guiyang 550025, China

**Keywords:** bryophyte, polar, adaptation, comparative genomic

## Abstract

The polar regions host a diverse array of moss species that have evolved to thrive in extreme environments. These mosses exhibit remarkable adaptations, including tolerance to freezing temperatures, desiccation, and ultraviolet radiation. Despite their ecological significance, genomic data on these organisms are still limited, impeding our understanding of their evolutionary history and adaptive mechanisms in the context of climate change. In this study, we present the first chromosome-scale genome assembly and annotation of the Arctic moss *Ptychostomum knowltonii*. The assembled genome is 408.8 Mb in size, anchored to 12 pseudochromosomes, with a scaffold N50 of 32.61 Mb. Repetitive elements account for 56.24% of the genome. The genome contains 28,014 protein-coding genes, with a BUSCO completeness of 96.20%. This genomic resource will enable future comparative genomic studies, enhancing our understanding of how polar mosses may respond to a warming climate and shedding light on their evolutionary trajectories in persistently extreme environments.

**Significance**

Genomic data from bryophytes provide a crucial foundation for investigating the vast array of resistance genes these organisms possess, making the sequencing and assembly of bryophyte genomes from various extreme habitats particularly valuable. In polar regions, mosses that are adapted to harsh conditions thrive, yet genomic data for these species remain limited. This gap in data hampers our understanding of their adaptive mechanisms in response to climate change. In this study, we present the genome assembly of the Arctic moss *Ptychostomum knowltonii*, which will enable future comparative analyses and provide essential insights into the adaptive strategies of mosses in extreme environments, as well as their responses to a changing climate.

## Introduction

Land plants consist of two sister lineages, i.e. vascular plants and bryophytes, and the latter comprise mosses, liverworts, and hornworts. Bryophytes are a group of minute and delicate creatures, with a haploid-dominant vegetative body (the gametophyte) and an unbranched sporophyte that remains attached to the gametophyte (Shaw and Renzaglia 2004). Their gametophyte is structurally simple with no vascular tissue and true roots (Ligrone et al. 2012). However, bryophytes have diversified significantly, with some 22,000 extant species (Christenhusz and Byng 2016). They have adapted to a wide range of habitats, including terrestrial, epiphytic, and aquatic environments, and are distributed across nearly all ecosystems globally (Degola et al. 2022). Bryophytes can endure a wide range of temperatures, from freezing cold to intense heat. They have evolved mechanisms to stabilize cellular structures during freezing and to reduce metabolic activity in high temperatures. These adaptations make them a promising source of novel resistance genes with potential applications in agriculture and biotechnology. However, compared with the significant progress made in genome sequencing of vascular plants, the number of sequenced bryophyte genomes remains limited, particularly for mosses adapted to extreme environments (Szovenyi et al. 2021).

To date, the Antarctic moss *Pohlia nutans* is the only polar bryophyte whose genome has been sequenced, providing insights into its adaptation to Antarctic conditions through mechanisms such as DNA repair, reactive oxygen species scavenging, and flavonoid biosynthesis (Liu et al. 2022). Like Antarctic plants, Arctic plants face highly challenging environments. In addition to short growing seasons, Arctic plants must cope with temperature fluctuations, strong winds, and nutrient-poor soils (Lee 2020), as well as disturbances from glacial meltwater (Kim et al. 2022). Therefore, sequencing the genomes of Arctic bryophytes is crucial for uncovering their adaptive mechanisms in response to these extreme conditions.

Here, we report the first chromosome-scale genome assembly of an Arctic moss, *Ptychostomum knowltonii* (Bryaceae, Bryales) (supplementary fig. S1, Supplementary Material online). *P. knowltonii* (formerly *Bryum knowltonii*) is a green or yellow-green monoecious (synoicous) moss widely distributed across various Arctic and sub-Arctic regions. It exhibits distinct morphological and ecological adaptations, making it a valuable subject for bryophyte research (Rolston 1982; Ramsay and Spence 2005). Furthermore, Byun et al. (2021) introduced the first genetic transfection method for Arctic moss species using *Bryum*. However, the lack of genomic resources has hindered further development of this technique. The availability of this *P. knowltonii* genome will enable future comparative genomic studies, facilitate genetic transfection research, and support the discovery and application of moss resistance genes. This will enhance our understanding of how mosses adapt to extreme environments, respond to a warming climate, and evolve in perpetually cold habitats.

## Results and Discussion

### Genome Assembly

**Fig. 1.**
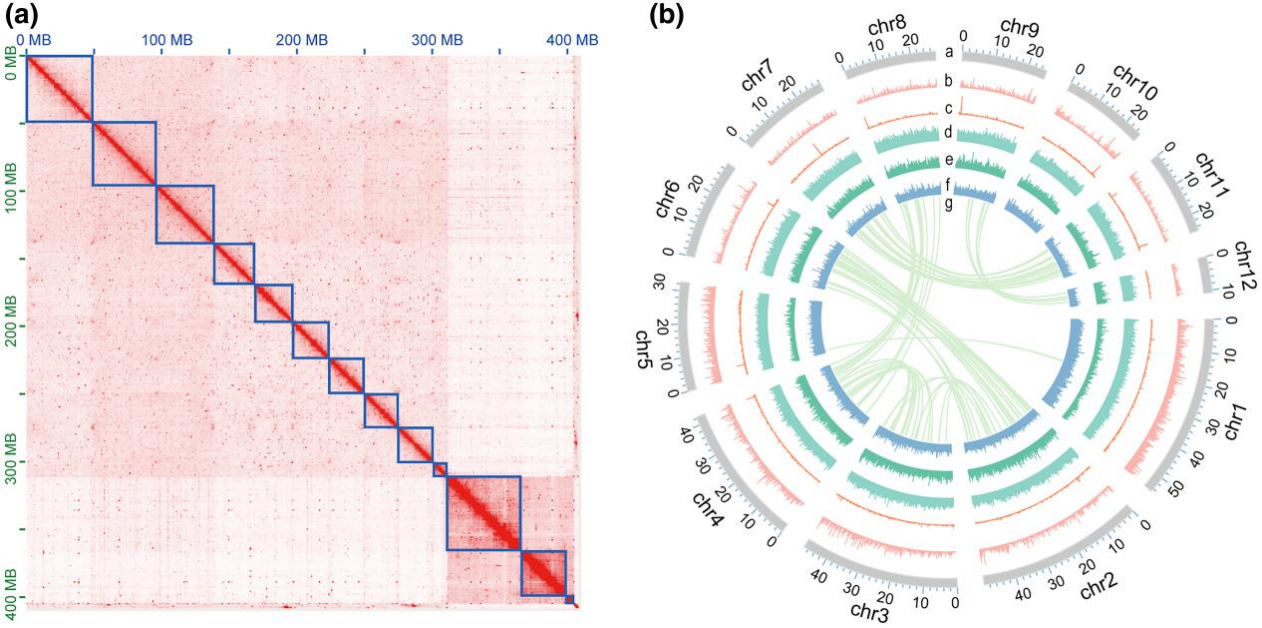
a) Heatmap of interaction frequency of genomic fragments of *P. knowltonii*. b) Circus plot of the genome of *P. knowltonii*. a, assembled chromosomes, b, *Gypsy* density, c, *Copia* density, d, repeat density, e, gene density, f, GC content, g, chromosome synteny.

To achieve a chromosome-level genome assembly of *P. knowltonii*, we generated 30.23 Gb of long-read data (∼74× coverage) using Oxford Nanopore Technologies for preliminary assembly, 55.88 Gb of Illumina clean short-read data (∼137× coverage) for genome survey and polishing, and 103.48 Gb of Hi-C clean data (∼253× coverage) for scaffolding. After removing redundancies, potential contaminants, and unanchored organelle fragments, we obtained an optimized genome assembly. The final assembly totaled 409 Mb in size, distributed across 692 contigs, with a contig N50 of 1.23 Mb and a scaffold N50 of 32.61 Mb (Table 1). The genome size of *P. knowltonii* is slightly smaller than the estimated 449 Mb based on *K*-mer analysis (see supplementary fig. S2, Supplementary Material online). Approximately 97.75% of the contigs (399 Mb) were anchored to 12 pseudochromosomes (Fig. 1a), with ambiguous “N” bases accounting for 282,110 bp, or 0.06% of the total genome length. The genome size of *P. knowltonii* is smaller than that of *P. nutans* (698.20 Mb), a species from the same family Bryaceae (Liu et al. 2022), but larger than *Bryum argenteum* (259 Mb) (Gao et al. 2023).

**Table 1:** Genome assembly and annotation statistics of *P. knowltonii*

Genome assembly	Value
Total scaffold length (bp)	408,794,622
Number of chromosome-level scaffolds	12
Longest scaffold (bp)	54,874,844
N50 of scaffold (bp)	32,607,604
Total contig length (bp)	408,512,582
Longest contig (bp)	6,478,272
N50 of contig (bp)	1,232,632
GC content (%)	40.48
BUSCO completeness (genome mode) (%)	92.20
**Genome annotation**	
Number of PCGs	28,014
Repeats in genome (%)	56.24
TEs in genome (%)	53.98
Mean coding sequence length (bp)	229.40
Average number of exons per gene	5.71
BUSCO completeness (protein mode) (%)	96.20
**Gene functional annotation**	
InterProScan (%)	56.27
Gene Ontology (%)	41.42
Kyoto Encyclopedia of Genes and Genomes (%)	62.49
Swiss-prot (%)	52.02
TrEMBL (%)	76.69
With *Arabidopsis thaliana* blast Hit (%)	56.22
Percentage of overall annotated genes (%)	79.72

To assess the quality of the *P. knowltonii* assembly, we performed a Benchmarking Universal Single-Copy Orthologs (BUSCO) analysis. Given that the embryophyta_odb10 database significantly underestimates BUSCO completeness in bryophytes (Bi et al. 2024), we used the viridiplantae_odb10 database instead. The results indicated that 92.20% of the gene space was captured in the *P. knowltonii* assembly, with 84.90% of the genes being complete and single-copy and 7.30% being complete and duplicated (Table 1 and supplementary fig. S3, Supplementary Material online).

### Repeat Annotation

We identified 229.89 Mb of repetitive sequences, representing 56.24% of the total genome length of *P. knowltonii*. The most abundant repeat elements were transposable elements (TEs), which accounted for 53.98% of the genome (Table 1). Among these, terminal inverted repeat transposons were the dominant type, comprising 29.13% of the genome, with *CACTA* elements contributing the largest proportion (supplementary table S1, Supplementary Material online). In contrast, the two long terminal repeat superfamilies, *Copia* and *Gypsy*, which are commonly abundant in angiosperms, were present at much lower levels in the *P. knowltonii* genome. Specifically, *Copia* elements accounted for 6.46 Mb (1.58% of the genome), while *Gypsy* elements contributed 26.20 Mb (6.41% of the genome) (supplementary table S1, Supplementary Material online).

### Gene Annotation and Functional Annotation

To predict protein-coding genes (PCGs) in *P. knowltonii*, we utilized two methods: BRAKER3 (Gabriel et al. 2024) and Helixer (Holst et al. 2023). The former predicted 15,718 genes, while the latter predicted 28,434 genes. Additionally, we incorporated gene predictions from homology alignments using miniprot (Li, 2023) and from transcriptome-based predictions using TransDecoder. These various sources of evidence were integrated using EVidenceModeler (Haas et al. 2008), resulting in a final set of 28,014 PCGs for *P. knowltonii* (Table 1), with an average gene length of 2,485 bp and an average of 5.71 exons per gene. However, the relatively small median gene length (1,781 bp) and intron length (185 bp) suggest that a subset of genes with very long introns has skewed the average gene length.

We evaluated the quality of the PCG set using the BUSCO protein model, which indicated a completeness of 96.20%. This is comparable to *P. nutans* (96.70%), higher than *B. argenteum* (94.80%), and slightly lower than the model bryophyte *Physcomitrium patens* (97.40%) (supplementary fig. S4, Supplementary Material online). Functional annotation assigned potential putative functions to 22,333 of the 28,014 PCGs in the *P. knowltonii* genome, representing 79.72% of the total gene set. The remaining 20.28% of the predicted genes lack functional annotations and may represent candidates for novel or previously uncharacterized functions.

### Sex Chromosome Characteristics

In the Hi-C heatmap of *P. knowltonii*, we observed that two chromosomes (chr1 and chr5) had lower interaction frequencies with the other 10 chromosomes (Fig. 1a). These chromosomes also showed lower gene density, higher GC content, and higher *Gypsy* density (Fig. 1b). This pattern suggests that the monoecious *P. knowltonii*, like *P. nutans*, may retain both male-specific (V) and female-specific (U) chromosomes in a single plant (Liu et al. 2022; Gao et al. 2023; Zhou et al. 2023). However, the evolution from U/V sex chromosome systems to co-sexuality may differ between these species. In *P. nutans* (22 chromosomes), this could be due to unreduced UV diploids, whereas in *P. knowltonii* (11 chromosomes), it may result from aneuploid UV haploids (Ekwealor and Roy 2023). Overall, the chromosome-level genome assembly of *P. knowltonii* may offer a valuable new model for studying sex system transitions and sex chromosome evolution in bryophytes.

## Materials and Methods

### Plant Materials, and DNA, RNA Extraction

Wild gametophytes of *P. knowltonii* were collected from Ny-Ålesund, Svalbard, Norway. The voucher specimen (collection number: Yao-002) has been deposited at the Herbarium of Fairy Lake Botanical Garden, Shenzhen, China. This plant material was regenerated in the lab by cultivating it in a low-temperature incubator for one month. Then, fresh shoots were cut, cleaned, and sterilized with a 2% NaClO solution for 10 s for producing tissue culture. The gem-free cultures were used for DNA extraction using the FastPure Plant DNA Isolation Mini Kit (Vazyme, Nanjing) and RNA-easy™ Isolation Reagent (Vazyme, Nanjing), respectively. DNA and RNA quantification and qualification was performed using 1% agarose gel electrophoresis, a Qubit fluorometer (Thermo Fisher Scientific, USA), and a NanoDrop 2000 spectrophotometer (Thermo Fisher Scientific, USA).

### Genome Assembly

We followed the method of Zhou et al. (2023) for constructing and sequencing Oxford Nanopore, Illumina, Hi-C, and RNA libraries. Low-quality sequences and adapter contaminations in the whole-genome sequencing data were filtered using Trimmomatic v.0.39 (Bolger et al. 2014). Based on the clean reads, we generated a *K*-mer depth distribution using Jellyfish v.2.3.1 (Marçais and Kingsford 2011) and estimated the genome size using GenomeScope v.2.0 (Ranallo-Benavidez et al. 2020). The *de novo* assembly of the *P. knowltonii* was carried out using Nextdenovo v.2.5.0 (Hu et al. 2024), and then the raw assembly was polished three times with NextPolish v.1.3.1 (Hu et al. 2019), using both the Nanopore long reads and cleaned Illumina short reads. Redundant contigs were removed with purge_dups v.1.2.6 (Guan et al. 2020), based on the Nanopore long reads and cleaned Illumina reads. To enhance the assembly’s contiguity, we employed the 3D-DNA pipeline (Dudchenko et al. 2017) to anchor the contigs to 12 pseudochromosomes. The anchored assemblies were then manually refined using Juicebox v.1.11.08 (Durand et al. 2016). For contigs that cannot be anchored to the 12 pseudochromosomes, we followed the process of Zhou et al. (2023) to remove contaminating contigs and organelle fragments.

### Repeat Annotation and Gene Annotation

Tandem Repeats Finder (TRF v.4.07) (Benson 1999) was used to identify tandem repeat sequences across the genome. For TEs, we applied the extensive *de novo* TE Annotator (EDTA v.2.0.1) pipeline (Ou et al. 2019), in combination with RepeatModeler2 (Flynn et al. 2020). To predict PCGs of *P. knowltonii*, we integrated three lines of evidence. Homology-based protein evidence was gathered from the proteome sequences of *P. patens* (Bi et al. 2024), *Marchantia polymorpha* (https://marchantia.info/), *Arabidopsis thaliana* (https://www.arabidopsis.org/), and Swiss-Prot (https://www.uniprot.org/). For transcriptome-based evidence, clean reads were aligned to the reference genome using HISAT v.2.2.0 (Kim et al. 2019) to generate BAM files. Gene model predictions were then made based on the soft-masked genome, protein evidence, and transcriptome data using the BRAKER3 pipeline v.3.0.6 (Gabriel et al. 2024), which incorporates AUGUSTUS (Stanke et al. 2006) and GeneMark (Brůna et al. 2020). We also performed *de novo* gene model predictions using Helixer v.0.3.3 (Holst et al. 2023), which leverages deep learning and hidden Markov models.

Additionally, transcriptome-based gene models were predicted using StringTie v.2.2.1 (Pertea et al. 2015) and TransDecoder v.5.5.0 (https://github.com/TransDecoder/TransDecoder). For additional homology-based evidence, we employed miniprot v.0.12-r237 (Li, 2023) to align the homology protein set to the genome. All these evidences were integrated using EVidenceModeler v.2.1.0 (Haas et al. 2008). Finally, Trinity v.2.8.4 (Haas et al. 2013) was employed for *de novo* assembly of transcripts; the gene models were updated and added untranslated regions and alternative splicing variants using the PASA pipeline v.2.5.3 (Haas et al. 2003). To assess the completeness of the gene space, the BUSCO v.3.1.0-based viridiplantae_odb10 (Manni et al. 2021) was employed. For gene function annotation, the annotated protein sequences were blasted against the UniProt (SwissProt and TrEMBL) and TAIR (https://www.arabidopsis.org/) mdatabases with an *E*-value cutoff of <1 × 10^−5^. The gene ontology annotations combine the results of eggNOG-mapper v.2 (Cantalapiedra et al. 2021), InterProScan v.5.51–85.0 (Jones et al. 2014), and PANNZER2 (Törönen et al. 2018), while the KEGG annotations combine the results of eggNOG-mapper v.2 (Cantalapiedra et al. 2021) and KofamKOALA (Aramaki et al. 2020).

## Supplementary Material

evae268_Supplementary_Data

## Data Availability

The raw sequencing data have been deposited in NGDC data center (https://ngdc.cncb.ac.cn/) under the project number PRJCA030700. Genome assemblies and annotations have been deposited in figshare repository at https://doi.org/10.6084/m9.figshare.27186099.
