# Supplementary material for: Chromosome-level Genome Assembly and Annotation of the Arctic Moss Ptychostomum knowltonii
Source: Genome Biol Evol. 2024 Dec 10;17(1):evae268. doi: 10.1093/gbe/evae268 (PMC11704416; doi:10.1093/gbe/evae268)
Supplement: evae268_Supplementary_Data [file evae268_supplementary_data.docx]

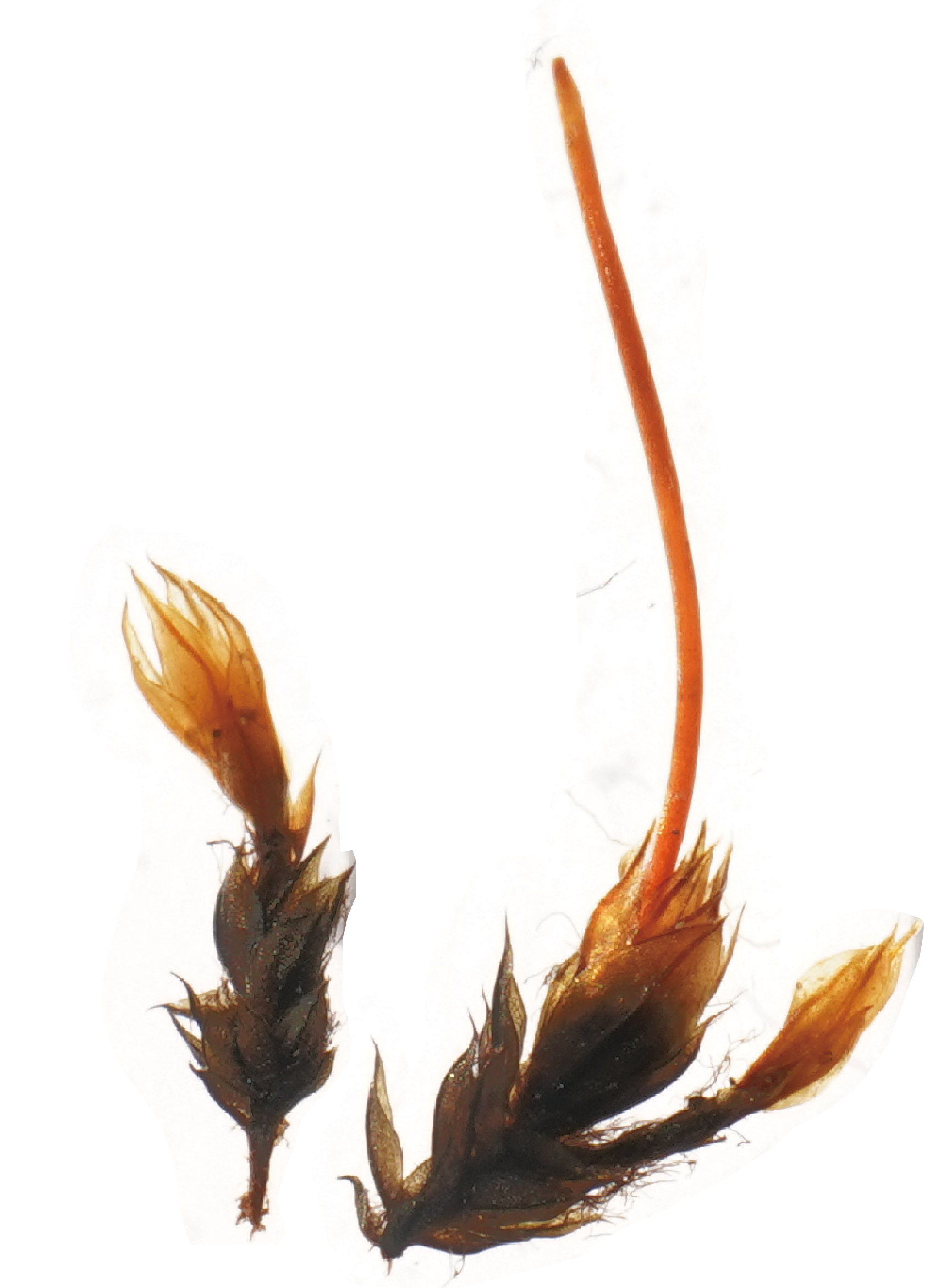


**Supplementary Figure S1. Plant morphology of *Ptychostomum knowltonii*.**

**
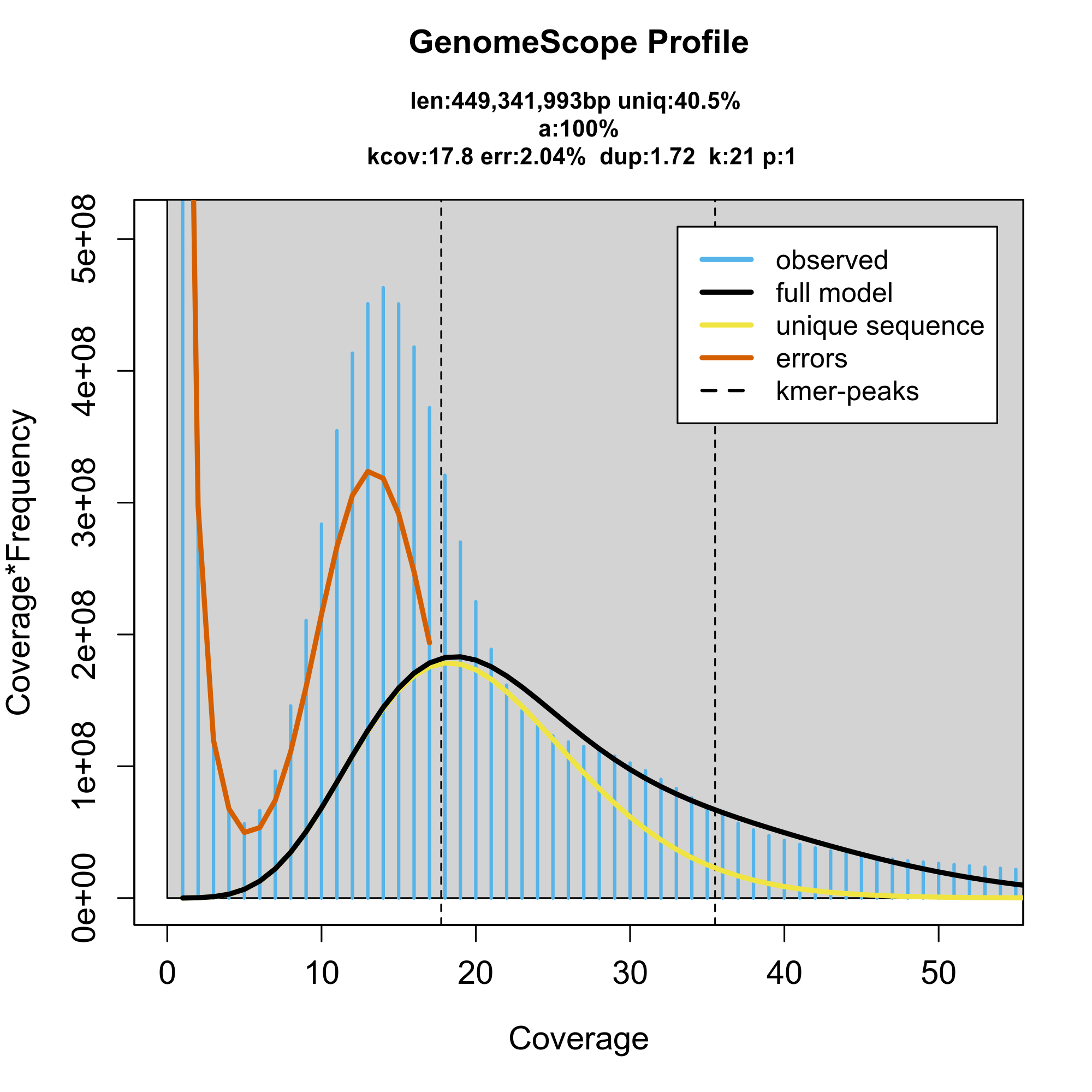
Supplementary Figure S2. 1 GenomeScope plots for *Ptychostomum knowltonii*.** The *K*-mer size is 21 and the estimated genome size is ca. 449.34 Mb.

**
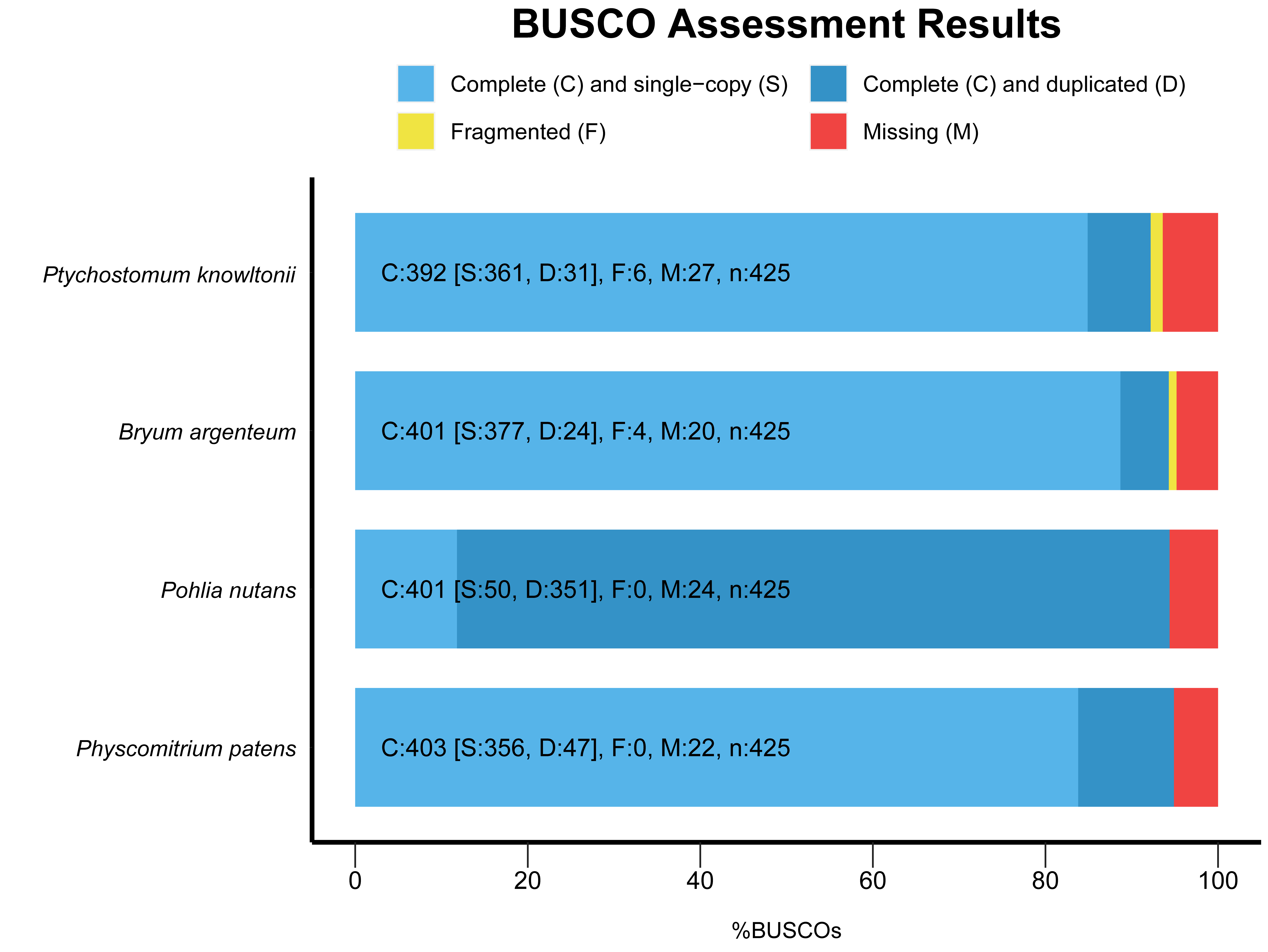
**

**Supplementary Figure S3. BUSCO assessment of genome assembly of *Ptychostomum knowltonii*, *Bryum argenteum*, *Pohlia nutans* and *Physcomitrium patens*.**


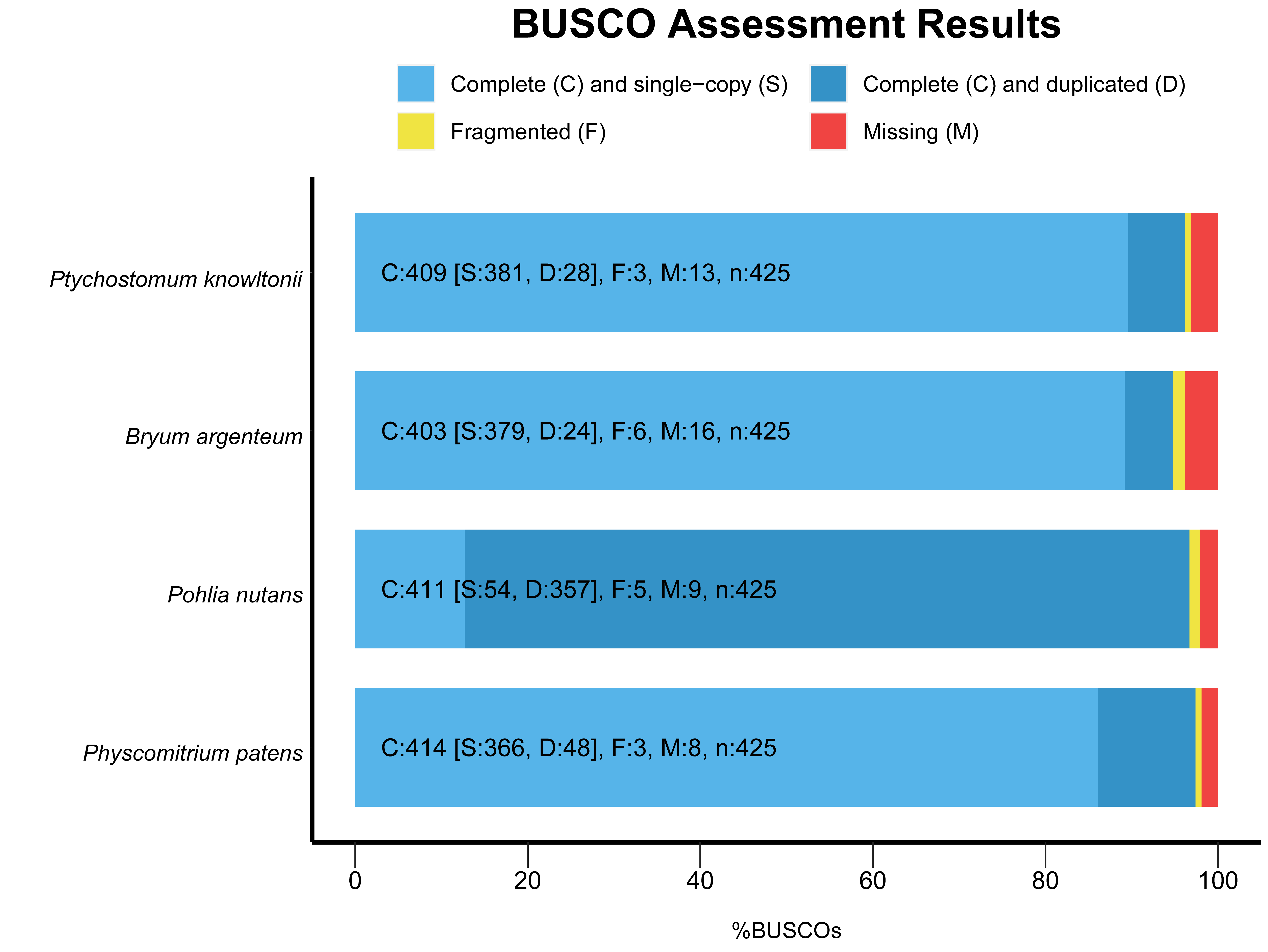


**Supplementary Figure S4. BUSCO assessment of protein-coding gene sets of *Ptychostomum knowltonii*, *Bryum argenteum*, *Pohlia nutans* and *Physcomitrium patens*.**

**Supplementary Table S1. Annotation and percentage of transposable elements of genome.**

| Class | Count | Length | % |
| --- | --- | --- | --- |
| DNA transposon | 371 | 155725 | 0.04 |
| LTR |  |  |  |
| Copia | 11261 | 6463335 | 1.58 |
| Gypsy | 26165 | 26199110 | 6.41 |
| unknown | 34032 | 16277540 | 3.98 |
| TIR |  |  |  |
| CACTA | 133774 | 47854305 | 11.71 |
| Mutator | 88167 | 31638004 | 7.74 |
| PIF Harbinger | 53362 | 17095727 | 4.18 |
| Tc1 Mariner | 27679 | 9607302 | 2.35 |
| hAT | 24578 | 12892440 | 3.16 |
| nonLTR |  |  |  |
| LINE element | 329 | 361198 | 0.09 |
| Penelope | 31 | 41329 | 0.01 |
| nonTIR |  |  |  |
| helitron | 67039 | 25336633 | 6.20 |
| repeat region | 67118 | 26587882 | 6.51 |
| Total | 533906 | 220510530 | 53.98 |
